# Association Between Family Support Combined With Exercise Rehabilitation and Psychological Resilience, Neurological Function and Daily Living Activities in Patients With Stroke and Anxiety

**DOI:** 10.62641/aep.v53i6.2063

**Published:** 2025-12-17

**Authors:** Can Ao, Lu Zhan, Yonghong Huang

**Affiliations:** ^1^Sports Department, Chongqing Jiaotong University, 400074 Chongqing, China; ^2^Department of Neurology and Geriatrics, The First Affiliated Hospital of Chongqing Medical and Pharmaceutical College, 400060 Chongqing, China; ^3^General Practice Department, The First Affiliated Hospital of Chongqing Medical and Pharmaceutical College, 400060 Chongqing, China

**Keywords:** family, exercise, stroke, anxiety disorders, psychological resilience, neurological function

## Abstract

**Background:**

To investigate the association between family-supported combined exercise rehabilitation and psychological resilience, neurological function and daily living activities in patients with stroke and anxiety.

**Methods:**

This retrospective study included 260 patients with stroke and anxiety disorder who attended The First Affiliated Hospital of Chongqing Medical and Pharmaceutical College between April 2022 and April 2024. On the basis of the intervention methods documented in their medical records, they were categorised into Groups A (*n* = 133) and B (*n* = 127). Group A received routine postoperative care plus exercise rehabilitation intervention, whereas Group B received the same intervention plus additional family support intervention. Both groups underwent a 12-week intervention period. Data for psychological resilience (Connor–Davidson resilience scale [CD-RISC]), anxiety (Hamilton Anxiety Scale [HAMA]), neurological function (National Institutes of Health Stroke Scale [NIHSS]), daily living activities (Barthel Index [BI]) and motor function (Fugl–Meyer Assessment of Motor Function [FMA]) scores at baseline, Week 6 and Week 12 were extracted from medical records. Repeated measures (Analysis of Variance [ANOVA]) was employed to compare time effects, between-group effects and interactions. Two-way ANOVA examined the interaction effect between family support and exercise rehabilitation. Multivariate linear regression analysis was performed by using scores at 12 weeks postintervention as the dependent variable, baseline scores as the independent variable and group assignment as the independent variable.

**Results:**

After 12 weeks of intervention, both groups showed increased CD-RISC, BI and FMA scores, along with decreased HAMA and NIHSS scores. Group B demonstrated superior improvement to Group A (*p* < 0.05). Repeated measures ANOVA revealed the significant main effects of time (*F* values ranging from 145.219 to 313.091 for each indicator, all *p* < 0.001) and between-group effects (*F* values ranging from 6.429 to 12.682 for each indicator, all *p* < 0.05). Significant interactions between time and group were observed for HAMA (*F* = 4.765, *p* = 0.009), NIHSS (*F* = 10.589, *p* < 0.001) and BI (*F* = 3.463, *p* = 0.032) scores, whereas no significant interaction was observed for CD-RISC (*F* = 0.728, *p* = 0.483) or FMA (*F* = 1.062, *p* = 0.335). Linear regression analysis indicated that after controlling for baseline scores, intervention group remained an independent predictor of change in all measures (*p* < 0.05).

**Conclusions:**

In patients with stroke and anxiety disorder, the combined intervention of family support and exercise rehabilitation is associated with improved psychological resilience; alleviated anxiety symptoms; and enhanced recovery of neurological function, daily living activities and motor function. Although the patient’s baseline severity of illness showed the strongest association with final recovery outcomes, family support interventions also constituted an independent favourable factor associated with improved recovery outcomes.

## Introduction

Stroke is a common acute cerebrovascular disease in clinical practice. It is characterised by multiple complications and high recurrence rates and mortality. It is currently a leading cause of death worldwide [1]. After experiencing a stroke, patients often face numerous challenges, including motor dysfunction. In addition to physical impairments, stroke can trigger psychological and mental disorders, leading to the development of anxiety disorders, which further hinder rehabilitation [2]. With the shift towards the bio–psycho–social medical model, stroke rehabilitation has become increasingly recognised to not solely focus on physical functional recovery. Psychological state and social support also play critical roles throughout rehabilitation.

Family support, as an important component of a patient’s social support system, has increasingly drawn attention for its role in the rehabilitation of patients with stroke and anxiety disorder. Families not only provide emotional support but also offer practical assistance in rehabilitation exercises and other activities [3, 4]. Research [5] has demonstrated that robust family support can improve psychological wellbeing, thereby enhancing quality of life. During rehabilitation training, patients’ confidence in their recovery can be enhanced by active assistance from their family members in exercises to improve their daily living abilities [6]. The effectiveness of exercise rehabilitation, as an important means of promoting the recovery of motor function in patients with stroke and anxiety disorder, has also been validated by clinical practice [7]. Through regular exercise training, muscle strength can be enhanced, joint mobility can be optimised and limb motor function can be improved [8].

However, current research on the synergistic effects of family support and exercise rehabilitation in the rehabilitation of patients with stroke and anxiety disorder remains relatively limited. In actual clinical practice, how to integrate family support and exercise rehabilitation resources well to leverage their respective advantages fully and promote the comprehensive physical and mental rehabilitation of patients is an urgent issue that needs to be addressed. This study aims to investigate thoroughly the effects of family support and exercise rehabilitation on psychological resilience, anxiety levels, neurological function, daily living activities and motor function in patients with stroke and anxiety disorder, as well as the interactive effects between the family support and exercise rehabilitation, to provide scientific evidence for the development of effective rehabilitation strategies in clinical practice.

## Materials and Methods

### Study Design

This retrospective study analysed the clinical data of 260 patients with stroke and anxiety disorder who attended The First Affiliated Hospital of Chongqing Medical and Pharmaceutical College between April 2022 and April 2024. The choices of patients and their families were respected within the scope permitted by the patient’s condition. Participants were grouped in accordance with their selected intervention methods: Group A received routine postoperative care plus exercise rehabilitation intervention, whereas Group B received an intervention that added home-based support intervention to the Group A regimen. Both groups underwent the interventions for 12 weeks. Inclusion criteria: (1) Meets the diagnostic criteria of “Diagnostic Points of Various Major Cerebrovascular Diseases in China 2019” [9] and diagnosed with stroke by Computed Tomography or Magnetic Resonance Imaging. (2) Meets the diagnostic criteria for anxiety disorders as outlined in the International Classification of Diseases, 11th Revision [10]. (3) Has a disease duration of 1–3 months and in the recovery period. (4) Clearly conscious and able to cooperate with the completion of scale evaluation and rehabilitation training. (5) Has complete clinical data. Exclusion criteria: (1) Has comorbidity with severe cognitive impairment, history of psychosis, or other psychiatric disorders. (2) Has comorbidity with severe cardiac, hepatic, renal and other vital organ failure. (3) Has complete loss of limb function that precludes motor rehabilitation. (4) Presence of communication disorders, such as severe aphasia and hearing impairment. (5) Has incomplete clinical data. This study was approved by the Ethics Committee of The First Affiliated Hospital of Chongqing Medical and Pharmaceutical College and is consistent with the Declaration of Helsinki. All participants signed informed consent forms.

### Treatment

Group A received standard neurology care plus exercise rehabilitation intervention, including condition monitoring, medication guidance and basic rehabilitation education. Vital signs and changes in neurological deficits were monitored daily, with the documentation of any complications. Standardised medications were administered on the basis of clinical condition. These medications included antiplatelet agents; circulatory enhancers; lipid-regulating drugs; and anxiolytics, such as diphenhydramine (Shijiazhuang Enbip Pharmaceutical Co., Ltd., National Drug Approval No. H20050299, Shijiazhuang, Hebei, China), atorvastatin (Huizhi Pharmaceutical Co., Ltd., National Drug Approval No. H20051408, Changzhou, Jiangsu, China) and escitalopram (Hunan Dongting Pharmaceutical Co., Ltd., National Drug Approval No. H20143391, Changde, Hunan, China). Drug effects, dosages and adverse reactions were explained. Basic rehabilitation knowledge education was provided as follows: By using verbal explanations and illustrated manuals, patients and families were educated on precautions during the stroke recovery phase, the importance of early rehabilitation training and guided simple joint movements to prevent deep vein thrombosis. The training frequency for exercise rehabilitation was five days per week. Weeks 1–4 involved foundational functional training, with each session lasting 30–45 min and including balance training and limb movement exercises. Weeks 5–12 featured advanced functional training, with each session extended to 45–60 min and incorporating coordination, gait and endurance training. A safety harness was worn during training, with therapists providing continuous supervision. Activity was immediately ceased if the patient experienced dizziness, palpitations, or other discomfort [11]. The intervention lasted 12 weeks.

Group B received home support interventions in addition to the care provided to Group A. During Week 1, rehabilitation therapists trained primary family members in psychological support techniques, such as listening to patients’ concerns, encouraging positive expression and avoiding negative language towards patients; daily care protocols for assisting patients with daily living activities, including eating, dressing and toileting; and key points for rehabilitation cooperation, such as learning correct postures for patient repositioning and passive joint mobilisation to prevent joint injury from improper handling. From Weeks 2 to 12, a rehabilitation nurse conducted biweekly home visits to assess family support implementation, provide on-site guidance for identified issues and adjust support plans in accordance with the patient’s rehabilitation progress. Concurrently, a family WeChat group was established to share weekly updates on the patient’s recovery, encourage experience exchange amongst family members and invite a psychologist to address emotional management concerns [12]. The synergy between family support and exercise rehabilitation was emphasised, as follows: Rehabilitation therapists synchronised daily exercise protocols with family members, instructing them to assist with home-based repetition. Family members documented the patient’s emotional responses during home training and relayed feedback to therapists, who then adjusted the following day’s training intensity accordingly. For instance, should anxiety arise from excessive difficulty, exercise complexity is temporarily reduced. The intervention lasted for 12 weeks.

### Observation Indicators and Evaluation Criteria

Baseline information, including gender, age, educational attainment, duration of the disease, type of stroke, number of stroke episodes and underlying medical conditions, were collected for all patients. All patient data recorded in the medical records system for the following indicators at baseline, six weeks postintervention and 12 weeks postintervention were extracted: psychological resilience, anxiety levels, neurological function, daily living activities and motor function. All scale data originated from archived assessment records within the medical records. Uniformly trained researchers extracted scale scores from the medical records to ensure consistency in data extraction, resolving any scoring discrepancies through a joint discussion mechanism.

The Connor–Davidson Resilience Scale (CD-RISC) developed by Connor and Davidson [13] was employed to quantify psychological resilience. Its total score ranges from 0 to 100, with high scores indicating high psychological resilience. The scale has a Cronbach’s alpha coefficient of 0.850 [14]. The Hamilton Anxiety Scale (HAMA) developed by psychiatrist Hamilton [15] was utilised to determine anxiety levels. This 14-item scale yields a total score ranging from 0 to 56, with high scores indicating severe anxiety symptoms. A total score ≥14 points is diagnostic for anxiety disorder. The scale demonstrates a Cronbach’s alpha coefficient of 0.790 [16]. The National Institutes of Health Stroke Scale (NIHSS) [17], developed by the NIH team, was applied to measure neurological recovery. This 11-item scale assesses consciousness, language, motor function and other domains, with a total score ranging from 0 to 42. High scores indicate severe neurological deficits, whereas low scores suggest neurological improvement. The Chinese version of this scale has a Cronbach’s alpha coefficient of 0.920 [18]. The Barthel Index (BI) developed by Dorothy Barthel and Florence Mahoney [19] was employed to evaluate daily living activities. This scale assesses 10 daily living activities. Its total score ranges from 0 to 100, with a high score indicating good functional capacity. The scale presents a Cronbach’s alpha coefficient of 0.901 [20]. The Fugl–Meyer assessment of motor function (FMA), developed by Fugl-Meyer *et al*. [21], was employed to measure motor function. This scale comprises 50 items for upper and lower limbs, with a total score ranging from 0 to 100. A high score indicates good motor function. The scale exhibits a Cronbach’s alpha coefficient of 0.973 [22].

### Statistical Analysis

Given that no missing data were encountered for any of the primary outcome measures or key baseline variables in this study, no data imputation was required, and the analysis was performed on the complete dataset. All data analyses were conducted by using IBM SPSS Statistics 27.0 (IBM, Armonk, NY, USA). The normality of continuous variables was assessed by employing the Kolmogorov–Smirnov test. Where variables met normality assumptions, they were expressed as mean ± SD, and intergroup differences were compared by using independent samples *t*-test. Nonnormally distributed data were represented as [M (Q_1_, Q_3_)] and analysed by using the Mann–Whitney U test. Categorical variables were presented as frequencies and percentages [n (%)] and analysed by conducting χ^2^ tests or Fisher’s exact tests. Repeated-measures Analysis of Variance (ANOVA) was utilised to analyse the time, between-group and interaction effects of intervention measures and time on each score, with the sphericity assumption tested. Where the sphericity assumption was not met, Huynh–Feldt correction was applied. Multivariate linear regression analysis was employed to investigate whether intervention groupings constituted independent predictors of outcome measures after controlling for baseline levels. Multivariate linear regression analyses were conducted by using scores on each scale at 12 weeks postintervention as the dependent variable, with intervention groupings and corresponding baseline scores as independent variables. Graphs and tables were generated with GraphPad Prism 10 software (GraphPad Software, La Jolla, CA, USA). *p* < 0.05 was considered statistically significant.

## Results

### Comparison of Baseline Data Between the Two Groups of Patients

In this study, the two patient groups were comparable in terms of gender; age; educational attainment; duration of illness; type of stroke; number of stroke episodes; underlying conditions; and preintervention CD-RISC, HAMA, NIHSS, BI and FMA scores, with no statistically significant differences observed (*p* > 0.05) (Table 1).

**Table 1.** **Comparison of baseline data between the two groups of patient**.

		Group A (*n* = 133)	Group B (*n* = 127)	Statistic	*p*	Cohen’s *d*
Sex, *n* (%)				χ^2^ = 0.134	0.714	
	Male	64 (48.12)	64 (50.39)			
	Female	69 (51.88)	63 (49.61)			
Age, year, mean ± SD		57.93 ± 9.88	57.43 ± 11.67	*t* = 0.374	0.709	0.047
Educational attainment, *n* (%)				χ^2^ = 0.763	0.683	
	Junior secondary school and below	68 (51.13)	71 (55.90)			
	Secondary school	43 (32.33)	39 (30.71)			
	College degree or above	22 (16.54)	17 (13.89)			
Disease duration, month, mean ± SD		4.82 ± 1.46	4.90 ± 1.50	*t* = 0.436	0.663	0.054
Stroke type, *n* (%)				χ^2^ = 0.353	0.552	
	Ischaemic	79 (59.40)	80 (62.99)			
	Haemorrhagic	54 (40.60)	47 (37.01)			
Number of stroke episodes, *n* (%)				χ^2^ = 0.439	0.508	
	First episode	112 (84.21)	103 (81.10)			
	Recurrence	21 (15.79)	24 (18.90)			
Hypertensive, *n* (%)		75 (56.39)	68 (53.54)	χ^2^ = 0.213	0.645	
Diabetes, *n* (%)		59 (44.36)	54 (42.52)	χ^2^ = 0.090	0.765	
CD-RISC scores, mean ± SD		43.23 ± 8.55	45.15 ± 7.94	*t* = 1.874	0.062	0.238
HAMA scores, mean ± SD		25.83 ± 5.18	26.48 ± 5.46	*t* = 0.985	0.326	0.122
NIHSS scores, mean ± SD		19.14 ± 3.87	20.04 ± 4.27	*t* = 1.782	0.076	0.224
BI scores, mean ± SD		46.05 ± 9.82	46.11 ± 9.07	*t* = 0.051	0.959	0.006
FMA scores, mean ± SD		42.05 ± 7.25	42.97 ± 7.51	*t* = 1.005	0.316	0.125

Note: CD-RISC, Connor–Davidson resilience scale; HAMA, Hamilton Anxiety Scale; NIHSS, National Institutes of Health Stroke Scale; BI, Barthel Index; FMA, Fugl–Meyer Assessment of Motor Function; SD, Standard Deviation.

### Comparison of CD-RISC Scores Between the Two Groups Before and After Intervention

**Fig. 1.**
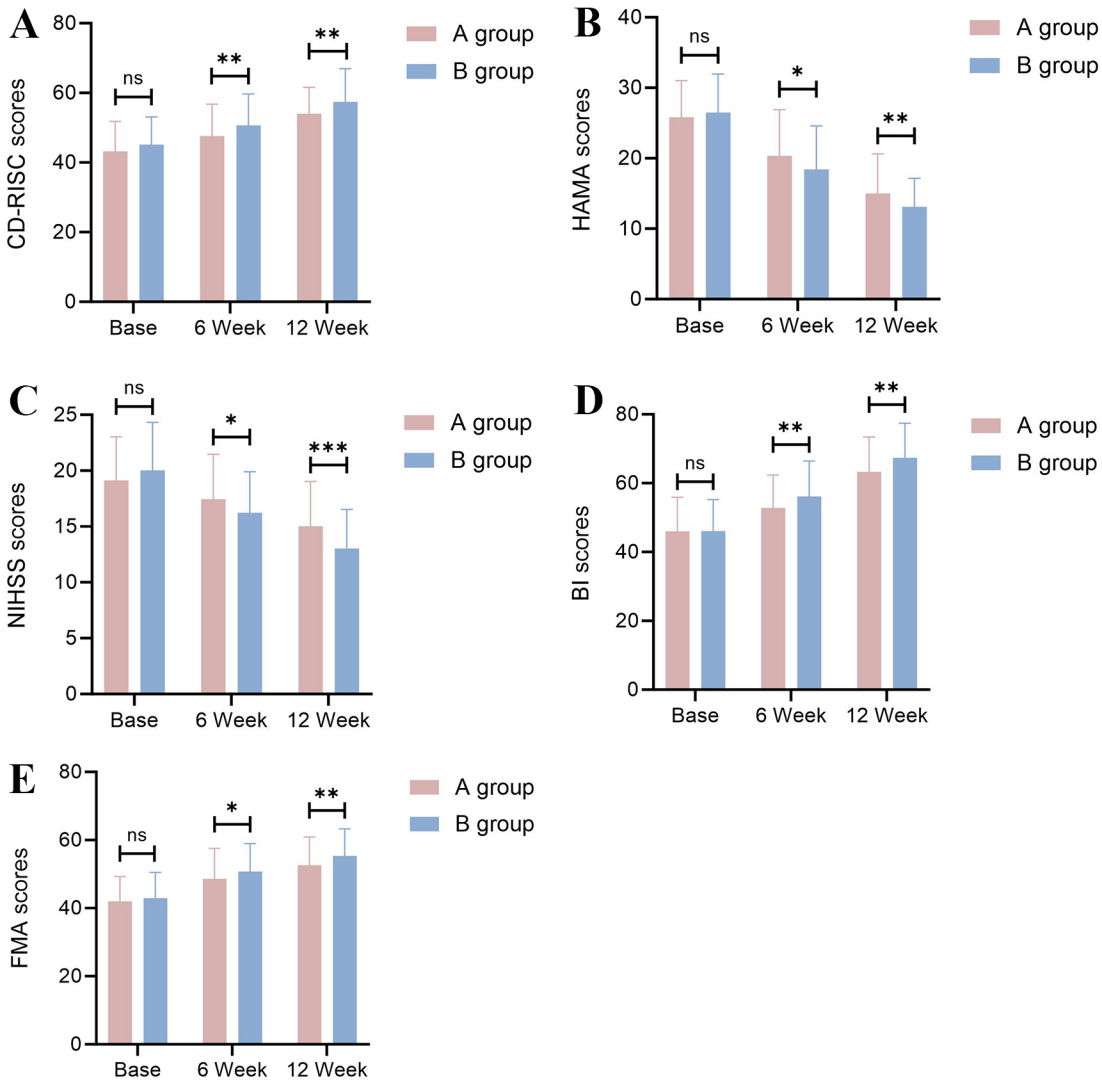
**Comparison of neurological function indicators before and after treatment**. (A) CD-RISC scores; (B) HAMA scores; (C) NIHSS scores; (D) BI scores; (E) FMA score (ns: not significant; *p < 0.05; **p < 0.01; ***p < 0.001). CD-RISC, Connor–Davidson Resilience Scale; HAMA, Hamilton Anxiety Scale; NIHSS, National Institutes of Health Stroke Scale; BI, Barthel Index; FMA, Fugl–Meyer Assessment of Motor Function.

In this study, CD-RISC scores did not significantly differ between the two patient groups prior to intervention (*p* > 0.05). After the 12-week intervention period, both groups exhibited an upwards trend in CD-RISC scores, with Group B demonstrating a significantly higher score (57.43 ± 9.50) than Group A (54.03 ± 7.57) (*p* = 0.002) (Fig. 1A).

### Comparison of HAMA Scores Between the Two Groups Before and After Intervention

In this study, HAMA scores did not significantly differ between the two patient groups prior to intervention (*p* > 0.05). After the 12-week intervention period, both groups exhibited a downwards trend in HAMA scores, with Group B demonstrating a lower score (13.10 ± 4.03) than Group A (15.03 ± 5.58) (*p* = 0.002) (Fig. 1B).

### Comparison of NIHSS Scores Between the Two Groups Before and After Intervention

In this study, NIHSS scores did not significantly differ between the two groups prior to intervention (*p* > 0.05). After the 12-week intervention period, both groups exhibited a downwards trend in NIHSS scores, with Group B demonstrating a lower score (12.02 ± 3.50) than Group A (15.04 ± 3.98) (*p* < 0.001) (Fig. 1C).

### Comparison of BI Scores Between the Two Groups Before and After Intervention

In this study, BI scores did not significantly differ between the two groups prior to intervention (*p* > 0.05). After the 12-week intervention period, both groups exhibited an upwards trend in BI scores, with Group B demonstrating a significantly higher score (67.34 ± 10.05) than Group A (63.29 ± 10.09) (*p* = 0.001) (Fig. 1D).

### Comparison of FMA Scores Between the Two Groups Before and After Intervention

In this study, FMA scores did not significantly differ between the two groups prior to intervention (*p* > 0.05). Following the 12-week intervention period, both groups exhibited an upwards trend in FMA scores, with Group B demonstrating a higher score (55.39 ± 7.88) than Group A (52.64 ± 8.21) (*p* = 0.006) (Fig. 1E).

### Repeated-Measures ANOVA for Each Outcome Index Between the Two Groups

The analysis of the main effect of time revealed statistically significant differences in scores across all five indicators at three time points: preintervention, six weeks postintervention and 12 weeks postintervention *(p* < 0.05). CD-RISC, BI and FMA scores showed an upwards trend over time, whereas HAMA and NIHSS scores exhibited a downwards trend. These trends are consistent across both groups. This finding suggests that, at the population level, patients demonstrated improved psychological resilience, daily living activity capabilities and motor function, alongside a reduction in anxiety levels and neurological deficits. The between-group main effect analysis revealed that after controlling for the time factor, Group B demonstrated significantly superior overall mean scores across all five indicators to Group A (*p* < 0.05). This result indicates that family support combined with exercise rehabilitation intervention programmes yield good outcomes at the group level. The interaction effect analysis showed a significant interaction effect between time and group assignment on HAMA, NIHSS and BI scores (*p* < 0.05). This finding indicates differing improvement trajectories between the two intervention groups regarding anxiety levels, neurological deficits and daily living activities, with Group B demonstrating superior progress to Group A. However, no significant interaction effect between time and group was observed for psychological resilience and motor function (*p* > 0.05) (Table 2).

**Table 2.** **Repeated measures analysis of variance for various indicators**.

Indicator	Time effect			Between-group effect			Interaction effect		
	*F*	*p*	Partial η^2^	*F*	*p*	Partial η^2^	*F*	*p*	Partial η^2^
CD-RISC	164.198	<0.001	0.398	12.682	<0.001	0.047	0.728	0.483	0.003
HAMA	313.091	<0.001	0.548	7.035	0.008	0.027	4.765	0.009	0.018
NIHSS	145.219	<0.001	0.360	6.429	0.012	0.024	10.589	<0.001	0.039
BI	284.968	<0.001	0.525	9.859	0.002	0.037	3.463	0.032	0.013
FMA	165.921	<0.001	0.391	8.244	0.004	0.031	1.062	0.335	0.004

Note: CD-RISC, Connor–Davidson resilience scale; HAMA, Hamilton Anxiety Scale; NIHSS, National Institutes of Health Stroke Scale; BI, Barthel Index; FMA, Fugl–Meyer Assessment of Motor Function.

### Linear Regression Analysis of Various Indicators Between the Two Groups

The results of linear regression analysis revealed that after controlling for respective baseline scores, the intervention group was an independent predictor of scores at 12 weeks for all measures (CD-RISC: *t* = 2.557, *p* = 0.011; HAMA: *t* = –3.422, *p* < 0.001; NIHSS: *t* = –6.196, *p* < 0.001; BI: *t* = 3.505, *p* < 0.001; FMA: *t* = 2.614, *p* = 0.009). For CD-RISC, BI and FMA, the unstandardised regression coefficients (B) for the groups were positive (B = 2.234, 4.036 and 2.205), indicating that at equivalent baseline levels, patients in Group B demonstrated significantly higher scores for psychological resilience, daily living activities and motor function at 12 weeks than those in Group A. For HAMA and NIHSS, the unstandardised regression coefficients (B) were negative (B = –2.047, –2.460), indicating that at equivalent baseline levels, patients in Group B exhibited significantly lower anxiety levels and neurological deficit scores at 12 weeks than those in Group A. Standardised regression coefficients (β) revealed that baseline scores were stronger predictors than intervention group assignment, although the latter’s predictive effect remained statistically significant (*p* < 0.05). This result indicates that while initial disease severity most profoundly influenced final rehabilitation outcomes, family support intervention also constituted an independent favourable factor promoting improved recovery (Table 3).

**Table 3.** **Results of linear regression analysis for each indicator**.

Indicator		*B*	*SE*	β	*t*	*p*	95% *CI*	R^2^	Adjusted R^2^
CD-RISC	Group	2.234	0.873	0.128	2.557	0.011	0.514–3.954	0.366	0.361
	CD-RISC-base	0.606	0.053	0.577	11.534	<0.001	0.503–0.709		
HAMA	Group	−2.047	0.598	−0.206	−3.422	<0.001	−3.226 to −0.869	0.076	0.069
	HAMA-base	0.183	0.056	0.196	3.268	0.001	0.073–0.294		
NIHSS	Group	−2.460	0.397	−0.316	−6.196	<0.001	−3.242 to −1.678	0.338	0.333
	NIHSS-base	0.498	0.048	0.524	10.268	<0.001	0.402–0.593		
BI	Group	4.036	1.152	0.196	3.505	<0.001	1.769–6.304	0.193	0.187
	BI-base	0.426	0.061	0.392	7.003	<0.001	0.306–0.546		
FMA	Group	2.205	0.844	0.135	2.614	0.009	0.544–3.866	0.318	0.313
	FMA-base	0.596	0.057	0.539	10.443	<0.001	0.483–0.708		

Note: CD-RISC, Connor-Davidson resilience scale; HAMA, Hamilton Anxiety Scale; NIHSS, National Institutes of Health Stroke Scale; BI, Barthel Index; FMA, Fugl–Meyer Assessment of Motor Function; CI, Confidence Interval; SE, Standard Error.

## Discussion

Stroke, as an acute cerebrovascular disease characterised by high rates of disability and recurrence, requires rehabilitation that addresses not only the restoration of neurological function and motor abilities but also the regulation of patients’ psychological states. This retrospective analysis of clinical data from 260 patients with stroke and anxiety systematically examined the effects of family support and exercise rehabilitation interventions on psychological resilience, anxiety levels, neurological function, daily living activities and motor function. Repeated measures ANOVA and linear regression analysis further revealed the interaction between interventions and time, as well as the independent predictive role of intervention groups. Its conclusions are as follows:

The present study revealed that CD-RISC scores significantly increased in both patient groups postintervention, with Group B exhibiting a greater improvement than Group A (*p* < 0.05). Repeated measures ANOVA indicated a significant main effect of time and a significant between-group main effect, although no significant interaction between time and group was observed. This outcome indicates that exercise rehabilitation is the primary factor in enhancing psychological resilience, whereas family support further amplifies the intervention’s efficacy. Mechanistically, exercise rehabilitation is associated with the activation of brain-derived neurotrophic factor secretion, neural remodelling around the infarct zone, increased endorphin release and anxiety alleviation. These changes may be correlated with enhanced positive cognitive attitudes towards recovery in patients [23, 24]. Aguiar *et al*. [25] demonstrated that regular exercise training enhances self-efficacy in patients with stroke, making them highly resilient in recovering from negative emotions. Although family support did not demonstrate a significant interaction effect, it may indirectly reinforce exercise’s impact on psychological resilience by providing sustained psychological security through empathetic listening and positive encouragement, thereby reducing feelings of frustration during training.

Anxiety is a common psychological disorder following stroke that can directly impede rehabilitation. This study found that HAMA scores decreased significantly in both groups over the intervention period. Group B exhibited significantly lower HAMA scores than Group A, with statistically significant main time, between-group and interaction effects (*p* < 0.05). This finding suggests that the combination of family support and exercise rehabilitation may be particularly effective in alleviating anxiety, highlighting the potential value of this integrated approach. The mechanism by which family support improves anxiety primarily manifests through emotional support. By assisting with daily activities and avoiding negative language, family members can reduce patients’ feelings of loneliness and helplessness, providing a sense of security that thereby reduces anxiety levels. Sharing experiences within WeChat groups can also alleviate family caregiving stress, indirectly preventing patients’ anxiety from worsening because of negative family emotions. Exercise rehabilitation, meanwhile, reduces the excessive activation of the hypothalamic–pituitary–adrenal axis, thereby decreasing cortisol secretion [26]. Furthermore, the functional improvements gained through exercise enhance patients’ sense of self-worth. The key to the synergistic effect lies in family support ensuring that patients feel secure during rehabilitation, thus reducing fear stemming from training difficulty, while the anxiety relief provided by exercise makes patients receptive to family support. Regression analysis revealed that the intervention group exerted a significant independent predictive effect on HAMA scores at 12 weeks. This result indicates that family support can reduce patients’ anxiety scores even when controlling for baseline anxiety levels. It suggests that the combined intervention of family support and exercise rehabilitation demonstrates superior association with improving patient outcome measures compared with exercise rehabilitation alone. Its clinical application value warrants further validation.

Neurological deficits constitute the primary cause of disability in patients with stroke. Changes in NIHSS scores within this study demonstrate that both groups exhibited a significant reduction in NIHSS scores over the intervention period. The scores of Group B were significantly lower than those of Group A, with a significant interaction effect between time and group allocation (*p* < 0.05). This outcome indicates that family support and exercise rehabilitation can synergistically promote neurological recovery. The improvement in neurological function through family support is correlated with treatment adherence. By learning the correct posture for passive joint mobilisation, family members can assist patients with daily basic training during home care, preventing rehabilitation interruptions due to the absence of healthcare personnel. Concurrently, the family supervision of medication administration and blood pressure monitoring reduces risk factors affecting neural repair, such as blood pressure fluctuations [27]. Through targeted training, exercise rehabilitation can directly improve the integrity of neural pathways, promote the functional reorganisation of the cerebral cortex and enhance the compensatory capacity of the contralateral hemisphere. This finding aligns with the conclusions of Xing and Bai [28]. Regression analysis revealed that the intervention group exerted a significant independent predictive effect on NIHSS scores at 12 weeks. The combined intervention of family support and exercise rehabilitation demonstrates practical clinical value.

The restoration of daily living activities is pivotal for the reintegration of patients with stroke into society. In this study, both groups exhibited a significant increase in BI scores over the intervention period. Group B demonstrated a significantly higher overall score than Group A, with a significant interaction effect between time and group (*p* < 0.05). This result indicates a synergistic effect between family support and exercise rehabilitation in enhancing daily living activities. Repeated measures ANOVA revealed significant main time effects, as well as between-group and interaction effects. This finding indicates that family support is a crucial factor in enhancing the ability to perform activities of daily living, while exercise rehabilitation lays the foundation for this by improving limb function. The direct impact of family support on BI manifests in caregivers integrating rehabilitation training into real-life scenarios during daily care. This situation enables patients to acquire practical skills within familiar environments, where immediate feedback markedly enhances training efficacy [29]. By contrast, although exercise rehabilitation did not demonstrate a main effect, it provides the physiological foundation for patients to perform daily activities by enhancing muscle strength and optimising joint range of motion. For instance, after exercise rehabilitation improves upper limb function, family assistance in practising independent feeding can rapidly elevate BI scores. Regression analysis indicates that the intervention group exhibits significant independent predictive power for BI scores at 12 weeks, demonstrating the practical clinical value of combined family support and exercise rehabilitation interventions.

The restoration of motor function constitutes the core objective of stroke rehabilitation. In this study, both groups exhibited significant increases in FMA scores over the intervention period. Group B demonstrated markedly higher overall scores than Group A (*p* < 0.05), although the interaction effect between time and group was nonsignificant (*p* > 0.05). This finding indicates that motor rehabilitation serves as the primary determinant in enhancing motor function, while family support amplifies its efficacy by consolidating training outcomes. Mechanistically, exercise rehabilitation serves as the primary driver of motor function improvement [30]. Balance and limb training during Weeks 1–4 enhances fundamental motor control capabilities, whereas coordination and endurance training during Weeks 5–12 reinforces neuromuscular pathway integrity, facilitating the precise cerebral regulation of limb movements. The synergistic effect with family support manifests as follows: family members assisting patients in repeating daily training content at home extends effective training duration and prevents motor memory decay. Furthermore, training discontinuation due to frustration is reduced by family members recording patients’ emotional responses during training and providing feedback to therapists for programme adjustments, ensuring rehabilitation continuity. Regression analysis revealed that the intervention group significantly and independently predicted FMA scores at 12 weeks. The lack of significant interaction effects may stem from motor function recovery being highly dependent on professional, standardised training protocols. Although family support extends training duration, it cannot fully replace standardised, hospital-based professional rehabilitation, thus failing to generate synergistic gains with exercise rehabilitation.

In summary, incorporating family support interventions alongside conventional exercise rehabilitation effectively enhances patients’ psychological resilience; reduces anxiety levels; and improves neurological function, daily living activities and motor function. The integration of family support into an exercise rehabilitation programme appears to enhance rehabilitation outcomes across multiple domains. The improvements observed in Group B suggest that family support adds substantial value to exercise rehabilitation alone, particularly for alleviating anxiety and improving neurological function and daily living activities. Although the severity of the baseline condition significantly impacts prognosis, the intervention of family support still demonstrates considerable clinical value. Therefore, in rehabilitation practice for patients with stroke and anxiety disorder, integrating family support with exercise rehabilitation to formulate multidimensional, individualised intervention strategies should prioritise the combination of family support and exercise rehabilitation. By fully leveraging the auxiliary role of family support through family training and home-based guidance, this approach holds positive value for comprehensively enhancing rehabilitation outcomes and quality of life.

This study represents the first instance in which rigorous repeated-measures ANOVA demonstrated a statistically significant interaction effect between two interventions within the specific population of patients with stroke and anxiety. It clearly identified the specific domains (HAMA, NIHSS and BI) in which this synergistic effect manifests, providing precise theoretical grounding for the development of composite rehabilitation programmes. This study not only examined psychological indicators (CD-RISC and HAMA) but also concurrently assessed neurological function (NIHSS) and objective physical function (FMA and BI). Linear regression analysis confirmed that family support constitutes an independent favourable factor, irrespective of baseline clinical condition. However, this study has certain limitations: Firstly, although baseline comparisons ensured comparability between groups, patient and family preferences for intervention methods may have influenced outcomes given that this work is a retrospective study, making completely avoiding selection bias impossible. Although assessors received standardised training and resolved discrepancies through discussion to control quality, formal interrater reliability testing was not conducted. Secondly, all patients were from a single centre, limiting sample representativeness. Thirdly, the 12-week intervention period did not assess the sustainability of long-term effects. Finally, the multivariable linear regression included only baseline scores and the intervention group, excluding confounding variables, which may introduce bias in estimating the intervention effect. The interaction between covariates and groups was not examined. The inability to identify heterogeneity in intervention effects across different patient subgroups may affect the precision of conclusions. Future studies should conduct multicentre, large-sample prospective randomised controlled trials incorporating additional confounding variables. These trials should enhance the consistency of quantitative assessments, extend follow-up periods to evaluate long-term outcomes and introduce objective measures to deepen mechanistic investigations. Furthermore, exploring digitally enabled home-based remote monitoring and exercise guidance models could improve intervention accessibility and standardisation, providing clinicians with intervention protocols with increased precision.

## Conclusions

Combined family support and exercise rehabilitation interventions can enhance psychological resilience; alleviate anxiety symptoms; and promote the recovery of neurological function, daily living activities and motor function in patients with stroke and anxiety disorder. Consequently, rehabilitation care for patients with stroke and anxiety should prioritise the integration of family support and exercise rehabilitation to elevate patients’ quality of life.

## Availability of Data and Materials

All experimental data included in this study can be obtained by contacting the corresponding authors if needed.
